# Identification and functional characterization of bidirectional gene pairs and their intergenic regions in maize

**DOI:** 10.1186/1471-2164-15-338

**Published:** 2014-05-05

**Authors:** Xiaoqing Liu, Xiaojin Zhou, Ye Li, Jian Tian, Qiuxue Zhang, Suzhen Li, Lei Wang, Jun Zhao, Rumei Chen, Yunliu Fan

**Affiliations:** Department of crop genomics and genetic improvement, Biotechnology Research Institute, Chinese Academy of Agricultural Sciences, Beijing, 100081 China; National Key Facility for Crop Gene Resources and Genetic Improvement (NFCRI), Beijing, 100081 China; School of life Science and Engineering, Southwest Technology University, Mianyang, 621000 China; Department of Agronomy, Agricultural University of Hebei, Baoding, 071000 China

**Keywords:** Genome-wide, Bidirectional gene pair, Bidirectional promoter, Maize

## Abstract

**Background:**

Bidirectional gene pairs exist as a specific form of gene organization in microorganisms and mammals as well as in model plant species, such as *Arabidopsis* and rice. Little is known about bidirectional gene pairs in maize, which has a large genome and is one of the most important grain crops.

**Results:**

We conducted a genome-wide search in maize using genome sequencing results from the inbred line B73. In total, 1696 bidirectional transcript pairs were identified using a modified search model. We functionally characterized the promoter activity of the intergenic regions of most of the bidirectional transcript pairs that were expressed in embryos using a maize embryo transient expression system. A comparative study of bidirectional gene pairs performed for three monocot (*Zea mays*, *Sorghum bicolor* and *Oryza sativa)* and two dicot (*Arabidopsis thaliana* and *Glycine max*) plant genomes showed that bidirectional gene pairs were abundant in the five plant species. Orthologous bidirectional gene pairs were clearly distinguishable between the monocot and dicot species although the total numbers of orthologous bidirectional genes were similar. Analysis of the gene pairs using the Blast2GO software suite showed that the molecular functions (MF), cellular components (CC), and biological processes (BP) associated with the bidirectional transcripts were similar among the five plant species.

**Conclusions:**

The evolutionary analysis of the function and structure of orthologous bidirectional gene pairs in various plant species revealed a potential pathway of their origin, which may be required for the evolution of a new species.

**Electronic supplementary material:**

The online version of this article (doi:10.1186/1471-2164-15-338) contains supplementary material, which is available to authorized users.

## Background

With the development of genome-sequencing techniques, increasing numbers of studies have reported the genome structure and organization—such as operons, gene clusters, and co-expressed genes—in diverse species [1–3]. One type of gene organization, head-to-head gene pairs, is an exception. Head-to-head gene pairs are defined in the human genome as being arranged on opposite strands with an intergenic region of less than 1.0 kb between the two transcription start sites (TSSs) [4]. Such a gene pair is termed a “bidirectional gene pair” (BDGP) and the intergenic region between the two TSSs is called a “bidirectional promoter” (BD PRO).

Studies on mammalian BDGPs started with the human genome. Adachi’s computational work defined the term and revealed that bidirectional gene (BDG) organization is a common architectural feature of the human genome. Subsequent studies on the BD PROs of human BDGPs provided additional insights [5]. Genome-wide analysis of the intergenic regions of BDGPs in higher vertebrates provided a foundation for optimized annotations of regulatory regions [6]. Other BD PROs were identified in the process of investigation of individual genes. Uwanogho et al. [7] described a BD PRO from the Mouse Recql4 and Lrrc14 BDGP. Bellizzi et al. [8] reported that expression of the human SIRT3 and PSMD13 gene pair is driven by a 788 bp BD PRO.

Although extensive investigation of BDGPs in mammalian and microbial genomes has been carried out, there are few publications regarding this phenomenon in plant genomes. Only four reports of individual BD PROs in model plants were published in the past 10 years. Keddie et al. [9] reported that a seed-specific *Brassica napus* oleosin promoter interacts with a G-box-specific protein and may be bidirectional; however, no subsequent reports on that promoter have appeared. Xie et al. [10] reported that fusion of a minimal promoter in the opposite orientation to the 5′ end of a polar promoter conferred bidirectional function upon the polar promoter in *Arabidopsis thaliana*. Moreover, the study showed that a cauliflower mosaic virus (CaMV) 35S minimal promoter could be bipolarized not only by fusion with another copy of the 35S minimal promoter in the reverse orientation, but also by fusion with an unrelated minimal promoter (*At*SAGmini) derived from a senescence-specific gene. Two reporter genes were expressed successfully in transgenic tobacco using an artificial BD PRO constructed from a 35S mini promoter fused to a natural polar promoter [11, 12]. The BD PROs in the intergenic regions between the *cab1* and *cab2*, *At5g06280* and *At5g06290* genes in *Arabidopsis thaliana* and the *Ocpi1* and *Ocpi2* genes in rice, and the *CaTin1* and *CaTin1-2* genes in hot pepper cultivar Bugang have been investigated [13–16]. To our knowledge, only three genome-wide analyses of plant BDGs have been conducted. Two focused on the *Arabidopsis thaliana* genome and the other was a comparative study of BDGs in the genomes of rice, *Populus*, and *Arabidopsis thaliana*[17–19].

The intergenic regions of BDGPs in microorganisms, mammals, and three plant species have been described; however, BD PRO activity was not detected *in vitro* or *in vivo* in the genome-wide studies of plants. In our study, we conducted a genome-wide survey in maize. In total, 1696 bidirectional transcript pairs (BDTPs) were identified using a modified search model. Using a maize embryo transient expression system, we functionally characterized the promoter activity of a large sample of the intergenic regions of BDTPs that were expressed in embryos. To confirm these results, we used a maize microarray data set to annotate BDTPs and functionally characterized the activity of a sample of these BD PROs. Based on two RNA-seq data sets (NCBI SRA accession number: SRA060791; SRA035621) generated from 21 DAP (days after pollination) and 25 DAP maize embryos, we obtained a group of BD PROs that can drive reporter gene expression in 20 DAP maize embryos transformed by particle gun bombardment. To assess whether the gene organization and expression pattern of endogenous genes driven by BD PROs was unique to maize or in common with other plant genomes, we analyzed four other plant genomes (*Arabidopsis thaliana*, *Glycine max*, *Oryza sativa*, and *Sorghum bicolor)* for which large-scale sequencing has been completed and found that the molecular functions (MF), cellular components (CC), and biological processes (BP) associated with the bidirectional transcripts (BDTs) were similar among the five plant species. Moreover, evolutionary analysis of the function and structure of orthologous BDTs in various plant species suggested both their origin and a potential pathway of their evolution.

## Results

### BDGPs are abundant in the maize genome and the majority encode proteins

In a previous study, the search model used to identify BD PROs in genome-wide searches defined the coordinates of the transcription start and end sites for each gene as the 5′-most and the 3′-most boundaries, respectively, of each gene cluster [5]. The distances between the 5′ ends of nearest-neighbor genes on opposite strands were then calculated. Intergenic regions that were less than 1.0 kb in length were considered to be BD PROs. Using this model we identified 678 BDGPs from 39656 genes in maize (Table 1; Additional file 1). However, these results did not contain a known BD PRO, a 635 bp intergenic region from the upstream of the translation start sits between the GRMZM2G368861_T02 and GRMZM2G368890_T01, whose presence was confirmed by further experiments.

**Table 1:** **Results of genome-wide searches for BDGPs in five plant species using a traditional search model**

Species	Genome size (Mb)	Total BDGPs	Total genes^a^	Total BDGs/Total genes (%)	Unique BDGs	Redundant BDGs^b^
**Maize**	2400	678	39656	3.4	1354	2
**Sorghum**	760	519	27609	3.76	1037	1
**Rice**	466	1874	55986	6.7	3740	8
**Soybean**	1115	399	46367	1.7	798	0
***Arabidopsis***	120	2527	27416	18.44	5016	38

^a^Total number of genes for each species.

^b^In cases where two BDGPs share one BDG, the shared BDG was counted twice in the total number of BDGs. Such BDGs are defined as redundant BDGs.

**Figure 1:**
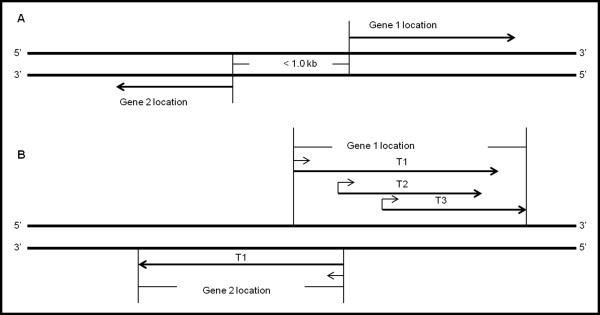
**Genome-wide search models. A**: Search model created by Trinklein. The gene location coordinates do not specify the location coordinates of different transcripts. **B**: Modified search model. This search model uses unique transcript location coordinates instead of unique gene location coordinates. Based on this modified search model, the intergenic region between the third transcript (T3) of gene 1 and the transcript of gene 2 can be considered to be a BD PRO. Such promoters would not be detected by the previous search model because the TSS of the first transcript (T1) of gene 1 would be considered the 5′-most boundary of the gene1 location coordinates that overlap with gene 2.

To avoid missing potential bidirectional genes such as those that give rise to two or more transcripts, we made a key modification to Trinklein’s search model. We used unique transcript location coordinates instead of unique gene location coordinates (Figure 1B). This change allows all transcripts located near a transcript on the opposite strand to be scanned, thereby dramatically increasing the number of candidate BDGPs. Using the modified search model, we identified 1696 BDTPs from 63541 maize transcripts (Table 2; Additional file 1). The known BD PRO that was missed using Trinklein’s search model was detected using our modified search model.

**Table 2:** **Results of genome-wide searches for BDTPs in five plant species using the modified search model**

Species	Genome size	Total	Total trans	Total BDTs/ Total	Unique	Redundant	Translated unique	New unique
	(Mb)	BDTPs	cripts^a^	transcripts (%)	BDTs	BDTs^b^	BDGs^c^	BDGs^d^
**Maize**	2400	1696	63541	5.34	2166	1226	1431	77
**Sorghum**	760	603	29448	4.1	1118	88	1118	81
**Rice**	466	2809	66338	8.46	4574	1044	3770	30
**Soybean**	1115	654	55787	2.3	1011	297	805	7
***Arabidopsis***	120	4386	35386	24.78	6627	2145	5062	46

^a^The total number of transcripts for each species.

^b^In cases where two BDGPs share one BDG, then the shared BDG was counted twice in the total number of BDGs. Such BDGs are defined as redundant BDGs.

^c^Because a single gene may produce multiple BDTs, the numbers of translated unique BDGs represent BDGs corresponding to non-redundant unique BDTs

^d^New unique BDGs are BDGs identified by the modified search model but not detected by the traditional search model.

To assess the relative effectiveness of the two search models, we compared the numbers of unique bidirectional genes (BDGs) and the overlap between the two sets of BDGs identified by the two search models. We removed two redundant BDGs from the 678 BDGPs identified using Trinklein’s search model leaving a total of 1354 unique BDGs (Table 2). The 1696 BDTPs identified using our modified search model corresponded to 3392 BDTs. A total of 2166 unique BDTs remained after removal of 1226 redundant BDTs (Table 3). The 2166 unique BDTs were derived from 1431 BDGs. Comparison of the 1354 BDGs identified using Trinklein’s search model with the 1431 BDGs identified using our modified search model showed that the former were a subset of the latter. Our modification of the original search model resulted in the identification of an additional 77 BDGs (Table 2).

**Table 3:** **Presence of TATA box and GC contents in BD/RD PROs from five plant species**

Species	BD PRO		RD PRO	BD PRO GC (%)	RD PRO GC (%)
	TATA box both ends^a^	TATA box one end^b^	TATA box one end		
**Maize**	0.1	8.1	6	49.9	44.5
**Sorghum**	0	4.4	5.2	55	46.5
**Rice**	0.4	9.5	6.5	48.2	44.3
**Soybean**	2.1	23.5	13.2	31	29.8
***Arabidopsis***	3.2	19.7	14.2	34	32.1

^a^Strict TATA box sequence detected on both ends of BD PRO.

^b^Strict TATA box sequence detected on either end of BD PRO.

RD PRO: Randomly obtained promoter regions extending 1000 bp upstream of the start ATG codon of each random gene from the genome of each species. Lengths of the RD PROs corresponded to the mean lengths of the BD PROs of each species. Mean promoter lengths: maize: 440 bp; sorghum: 440 bp; rice: 540 bp; soybean: 570 bp; Arabidopsis: 460 bp.

There were three categories of BDGPs: two coupled protein-coding genes, and one protein-coding gene coupled with a pseudogene or one protein-coding gene coupled with a transposable element (Additional file 2). Among the 2166 unique BDTs identified in maize, 2143 corresponded to BDGPs in which both genes encoded proteins. The remaining 13 BDGPs corresponded to 13 protein-coding genes that were each coupled to one transposable element or to 1 of 12 pseudogenes. It is interesting that 42.1% of BDGPs were distributed on chromosomes 1, 2, and 3 and that 34.9% of the BD PROs are 100–300 bp in length (Additional file 3).

These results suggest that the modified search model is more effective than the previous one and that the form of gene organization in which a BD PRO drives the expression of two flanking genes is abundant in the maize genome.

### Low frequency of strict TATA box sequences and high GC content in BD PROs in maize

Investigation of the structure and sequence characteristics of BD PROs may further our understandings of BDGPs. BD PROs are different from polar directional promoters. Previous studies showed that most BD PROs lack a TATA box. Only 8% of BD PROs contained a strict TATA box on either strand, but 28% of polar directional promoters contained a TATA box on the forward strand [5]. A similar report showed that only 9% of BD PROs contained a TATA box, compared to 29% of polar directional promoters [20]. Our findings are consistent with these previous results in that only 0.1% of the BD PROs in maize contained a strict TATA box (TTATTT/TATAAAT/TATTAAT/TATATAA) on both strands (Table 3). In a set of 1000 random polar directional promoters (RD PROs) from maize with lengths corresponding to the mean length of the BD PROs, 6.0% contained a TATA box on one strand (Table 3). A strict TATA box was present on one or the other strand in only 4.1% of the BD PROs in maize. These results indicate a bias against TATA boxes in BD PROs.

High GC content is another characteristic of BD PROs. Human, rice, *Arabidopsis*, and *Populus* BD PROs were shown to have a higher GC content than random promoters [4, 5, 17, 21]. The BD PROs identified in this study had a GC content of 49.9%, compared to 44.5% for the RD PROs (Table 3). Our findings on the GC content of BD PROs in maize are consistent with the results of the previous studies.

### Functional analysis of BD PROs in maize

To verify that the intergenic regions in BDGPs were functional as BD PROs and produced transcripts, we used a maize microarray (MA) expression data set (GEO accession number: GSE54310) and two RNA-seq (RS) data sets (NCBI SRA accession numbers: SRA060791; SRA035621) to annotate all BDGPs (Additional file 2). Based on relative expression values from 21 and 25 DAP embryo data from the MA and RS data sets (Additional file 2), we cloned 9 and 18 putative BD PROs, respectively, from the maize inbred line B73. These promoters were designated MA-1…MA-n and RS-1…RS-n. We also randomly cloned 129 putative BD PROs from genome-wide search (GS) results that were designated GS-1… GS-n. All of the promoters were cloned into a GFP/GUS dual reporter gene vector derived from pCAMBIA3301 (see Methods). We used particle bombardment of 20 DAP immature maize embryos as a transient expression system to identify the functions of the putative BD PROs. GFP expression in embryos was detected using an epifluorescence microscope and GUS expression in embryos was detected by histochemical staining.

We used MA data to annotate all of the BDGPs in maize. Only 564 genes and 74 BDGPs are covered by the Affymetrix maize microarray because the 17555 microarray probes covered only about 13339 of the genes in maize. (Additional file 2). The existence of expression data for the 74 BDGPs covered by the microarray demonstrated that the genes were transcribed. Subsequently, we analyzed the two RS data sets to characterize the transcripts of all the BDGPs in maize further. The RS data sets (Additional file 2) showed that the transcripts from most of the BDGPs were detected in 21 and 25 DAP embryos. However, a few BDTPs were not detected, possibly due to stringent tissue-specific expression patterns that did not include the embryo.

**Figure 2:**
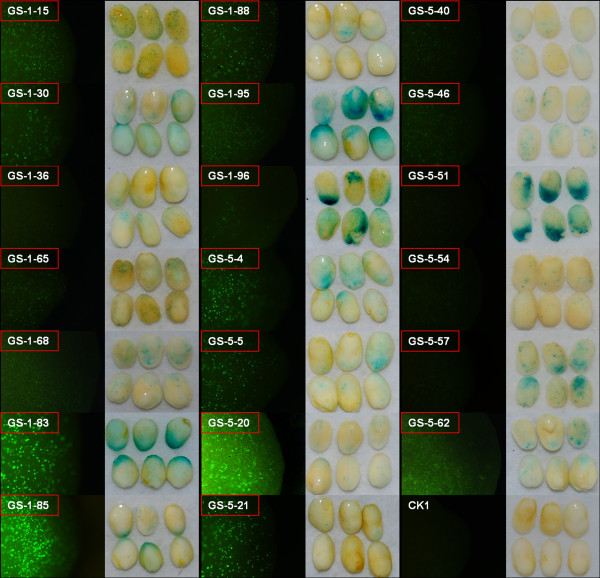
**Identification of putative GS BD PROs.** Embryos were isolated from 20 DAP immature maize kernels. Each construct was introduced twice by particle gun bombardment with six embryos per bombardment. GS-n represent the promoters cloned randomly from 1696 putative BD PROs; CK: Embryos bombarded with empty vector plasmid. Putative BD PROs that showed promoter activity in both directions are indicated by a red box.

**Figure 3:**
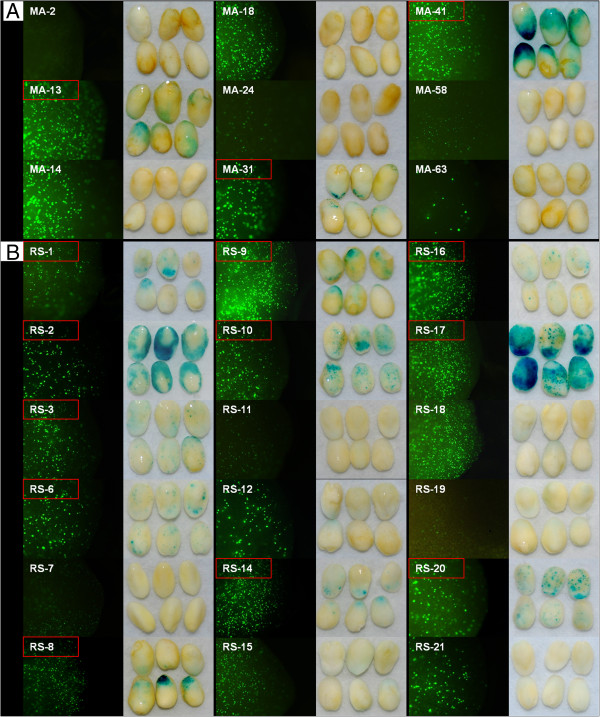
**Identification of BD PROs from the MA and RS data sets.** Embryos were isolated from 20 DAP immature maize kernels. Each construct was introduced twice using a particle gun with six embryos per bombardment. **A**: Promoters cloned based on the microarray data set (MA); **B**: Promoters cloned based on the RNA-seq data set (RS). Putative BD PROs that showed promoter activity in both directions are indicated by a red box. The remaining putative BD PROs showed either promoter activity in one direction or no promoter activity.

Although the above results confirmed the existence of transcripts associated with the BDGPs, they did not demonstrate that the transcripts were products of the intergenic regions acting as promoters driving transcription in both directions. To verify that expression of the annotated BDGPs was promoted by the putative BD PROs, we randomly cloned 129 promoters from among 1696 putative BD PROs identified in the genome-wide search (GS BD PROs). Using the transient transformation assay, we identified 20 functional BD PROs with promoter activity in both directions (Figure 2). Seventy-eight putative GS BD PROs had promoter activity in only one direction and thirty-one putative GS BD PROs showed no activity in either direction (Additional file 4). To confirm these results, we selected nine BDGPs from the MA data set and 18 BDGPs from the RS data sets that had high relative expression values in 20 and 25 DAP maize embryos (Additional file 2) as candidates to clone BD PROs. The transient transformation assay results showed that three of the candidates from the MA data set and 11 of the candidates from the RS data set functioned as BD PROs (Figure 3). Five of the MA candidates and seven of the RS candidates were transcribed in only one direction. One of the MA candidates showed no promoter activity in either direction (Figure 3). These results demonstrated the existence of transcripts from BDGPs in maize and that the intergenic regions between them function as BD PROs.

### Functional classes of BDTs in maize

**Figure 4:**
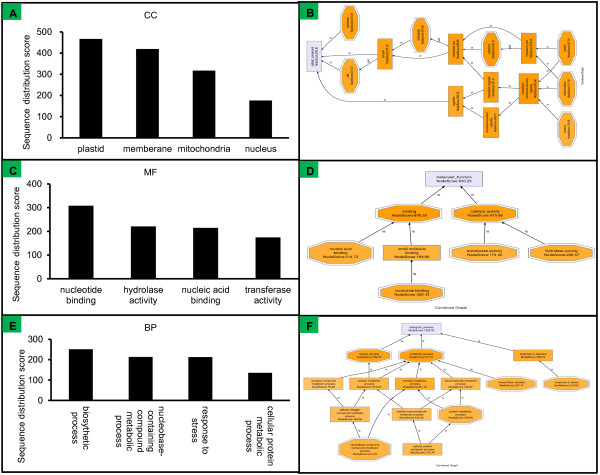
**Classification of the characteristics of the BDTs of maize. A, C**, and **E**: Multi-level pie charts of cellular components (CC), molecular functions (MF), and biological processes (BP) respectively. **B, D**, and **F**: Combined graphs of CC, MF, and BP respectively. Combined Graphs were generated by filtering the annotated sequences using the node sequence filter (>10% of the total number of sequences) and score filter (>100) together. Parent nodes that did not annotate more sequences than their children terms were omitted from the graph. Multi-level pie charts were derived from Combined Graphs that displayed only the lowest GO terms per branch. Node coloring by score value highlights the areas in which annotations are most concentrated.

The previous studies pointed out that the BDGPs/BDTPs may be associated functionally in vertebrates [5, 22] and plants [17, 18]. To test this hypothesis in maize, we used the bioinformatics software Blast2GO to functionally annotate the maize BDT sequences [23]. The annotation allowed us to assign the BDTs into three functional classes: molecular functions (MF), cellular components (CC), and biological processes (BP). The advantage of the Blast2GO is to visualize the functional information on the GO direct acyclic graph (DAG), and displayed by the Combined Graph. The colors nodes consider the places of direct annotation. To obtain a compact representation of the information of BDTs, we set the sequence filter to 10% which means only those nodes with at least 10% of the total number of sequences assignments will be displayed, and set the score filter to 100, additionally, which means parent nodes that do not annotate more sequences than their children terms will be omitted from the graph. The Combined Graph of the CC, MF and BP of maize BDTs are showed by the Figure 4B,D and F, respectively. These parameters finally led to most concentrated DAG with detailed sequence annotations. This graph could provide more information about the most concentrated function of the BDTs, however, in order to provide an optimal view of the dataset’s most relevant terms, the multi-level pie [24] was employed to ‘cut’ the GO DAG locally at different levels which only display the lowest GO terms per branch by stetting the same sequence filter to 10%. According to the multi-level pie showed in figure A, C and E, we can clearly found that the CCs most highly associated with the BDTs were plastids, membranes (plasma membrane), mitochondria, and nucleus (Figure 4A), and the nucleotide and nucleic acid binding, transferase activity, and hydrolase activity were the most relevant MFs (Figure 4C), and the biosynthetic processes, nucleobase-containing compound metabolic processes, cellular protein metabolic processes and stress responses were the most relevant BPs.

The distribution of BDT functions among specific CCs, MFs, and BPs suggests that the associations of BDTPs with functional classes are not random. They are associated with a relatively limited set of CCs, enzyme activities, and biological processes suggesting that they play an important role in maize.

### Comparative analysis of BDGP features in five plant genomes

BDGPs have been investigated extensively and are a common feature of human, yeast, and vertebrate genomes [4, 6, 25]. Two studies described BDGPs in the *Arabidopsis*, rice, and *Populus* genomes but did not compare the features of the BDGPs with those of other plant genomes [17, 18]. Hence, we chose two monocot species, rice and sorghum, and two dicot species, *Arabidopsis* and soybean, to attempt to identify common features of BDGPs across an evolutionarily diverse group of plant species.

To determine if BDGPs/BDTPs are a common feature of plant genomes, we used both the Trinklein search model and our modified search model to identify BDGPs/BDTPs in the four plant genomes. We also detected other similar types of gene organization, including convergent gene pairs (CGPs), for which the distance between the 3′ ends of the two genes is less than 1.0 kb, and co-direction gene pairs (CDGPs), for which the distance between the 3′ and 5′ ends of the two genes on the same strand is less than 1.0 kb. All species tested showed positive results (Additional files 1). BDGs and BDTs represented 1.7–18.44% of the total genes and 2.3–24.78% of total transcripts, respectively (Tables 1 and 2). We also found that CGPs and CDGPs were widespread in plant genomes (Additional file 3). Mapping the BDTPs to the chromosomes in each species showed that chromosome 1 in rice, sorghum, and *Arabidopsis*, and chromosome 8 in soybean had the highest number of BDTPs. Other chromosomes with high numbers of BDTPs included chromosomes 2, 3, and 4 in sorghum, chromosomes 2 and 3 in rice, chromosomes 3 and 5 in soybean, and chromosome 6 in *Arabidopsis* (Additional file 3). These results indicated that BDGPs/BDTPs are a common feature of plant genomes. Comparison of the sets of BDGs identified by the Trinklein search model with those identified using our modified search model showed that the former were subsets of the latter and that the modified search model identified 81, 30, 7, and 46 additional BDGs in sorghum, rice, soybean, and *Arabidopsis*, respectively (Table 2). These results further confirmed the efficacy of the modified search model.

Our sequence analysis of BD PROs in maize revealed a very low frequency of strict TATA box sequences on either strand, a high GC content, and 34.9% of BD PROs with lengths of 100–300 bp, consistent with previous reports [5, 20]. We analyzed BD PRO sequences from sorghum, rice, soybean, and *Arabidopsis* to determine if these three characteristics are also common features of BD PROs in other plant genomes. A strict TATA box was present on both strands in 0.4%, 2.1%, and 3.2% of BD PROs in rice, soybean, and *Arabidopsis*, respectively (Table 3). No BD PROs in sorghum contained strict TATA box sequences on both strands. A strict TATA box was present on either strand in 4.4%, 9.5%, 23.5%, and 19.7% of BD PROs in sorghum, rice, soybean, and *Arabidopsis*, respectively (Table 3). Only 2.2%, 4.8%, 12.8%, and 9.8% of the BD PROs contained a strict TATA box on either strand in sorghum, rice, soybean, and *Arabidopsis*, respectively. A TATA box was present on one strand in 5.2%, 6.5%, 13.2%, and 14.2% of 1000 RD PROs in sorghum, rice, soybean, and *Arabidopsis*, respectively (Table 3). These results suggest a bias against strict TATA box sequences in the BD PROs of these four species, similar to that in maize. The BD PROs of sorghum, rice, soybean, and *Arabidopsis* all had higher GC contents than the sequences of RD PROs, consistent with the results from maize (Table 3). We found that BD PRO lengths in all five plant species were predominantly 100–300 bp, which is consistent with BD PRO lengths in the human genome [5]. We also found relatively high proportions of BD PROs less than 400 bp in length in sorghum and greater than 600 bp in length in rice and soybean (Additional file 3). The BD PROs and RD PROs of all three monocot species had higher GC contents and contained fewer TATA box sequences than those of dicot species. This phenomenon is similar to the GC3 codon bias between monocot and dicot plants [26].

Based on functional annotation using Blast2GO, we showed that BDTPs play important roles in maize. We also analyzed the functional classes of the BDTPs in sorghum, rice, soybean, and *Arabidopsis*. BDTPs in sorghum, rice, and *Arabidopsis* were functionally associated with mitochondria using a stringent score filter value, and were associated with the nucleus in all five species using a non-stringent score filter value (Figure 4A,B and Additional file 5). Catalytic activity and binding activity were the primary molecular functions associated with BDTPs (Figure 4C/D and Additional file 5). Catalytic activity included transferase and hydrolase activities and binding activity included DNA, RNA, nucleotide, and protein binding. We found that BDTs were functionally involved in metabolic processes, cellular processes, developmental processes, and responses to stimulus in all five plant species (Figure 4E and F and Additional file 5). Reduction of the sequence filter value to > 1% (data not shown) revealed functional associations with metabolic processes including DNA metabolic processes, translation, cellular protein modification, lipid metabolic processes, and carbohydrate metabolic processes; signal transduction involved in major cellular processes; developmental processes including flower and embryo development and morphogenesis; and responses to external stimuli, including abiotic stress, biotic stress, and environmental factors. Transport processes appeared as biological processes with high scores only in rice and *Arabidopsis*.

These results indicate that bidirectional gene organization is a common feature in plant genomes. BD PROs in plants have less strict TATA box sequence requirements and a high GC content. Based on comparative analysis of the functional associations of BDTPs with CCs, MFs, and BPs, the patterns of bidirectional gene participation in metabolic processes are similar in the five plant species and, therefore, may play important roles in plants.

### Orthologous transcripts of BDTPs reveal a potential pathway for their evolution

Evolutionary conservation of orthologs usually indicates functional importance. We investigated orthologous transcripts corresponding to the unique BDTs of 1696 maize BDTPs in sorghum, rice, soybean, and *Arabidopsis* (Table 4). We mapped 2166 unique maize BDTs to the target four plant genomes and 1781, 1716, 1573, and 1543 orthologous transcripts were identified in sorghum, rice, soybean, and *Arabidopsis*, respectively. To investigate structural characteristics, we calculated the number of conserved gene pairs with distances of 0.5, 1.0, 2.0, and 5.0 kb between the TSSs of all of the identified orthologous transcripts. There were 602 and 1029 BDGPs in the monocot species sorghum and rice, respectively, and 79 and 10 BDGPs in the dicot species soybean and *Arabidopsis*, respectively, suggesting that BDGPs/BDTPs are functionally conserved but structurally diverse among plant genomes.

**Table 4:** **Genes orthologous to maize BDTPs in four other plant species**

Species	Total	Distance between TSSs of gene pairs			
		0.5 kb	1 kb	2 kb	5 kb
**Sorghum**	1,781	506	602	734	886
**Rice**	1,716	833	1,026	1,130	1,439
**Soybean**	1,573	73	79	100	111
***Arabidopsis***	1,543	9	10	14	41

In total, 2166 unique BDTs from 1696 maize BDTPs were used to search for orthologous transcripts.

**Figure 5:**
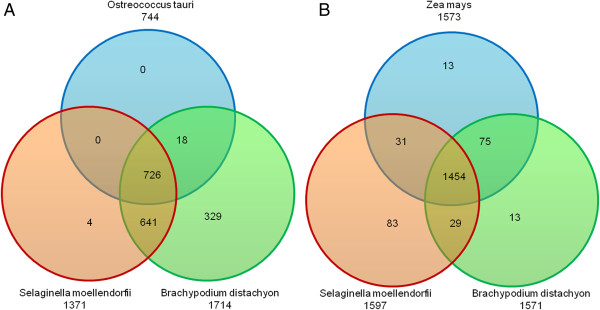
**Venn diagram of the distribution of orthologous transcripts of maize BDTs shared among plant species. A**: Analysis of orthologous transcripts of maize BDTs in *Brachypodium distachyon*, *Selaginella moellendorfii*, and *Ostreococcus tauri*. **B**: Analysis of orthologous transcripts of *Ostreococcus tauri* BDTs in *Brachypodium distachyon*, *Selaginella moellendorfii*, *Ostreococcus tauri*, and *Zea mays*. Numbers under the species names represent the total numbers of mapped orthologous transcripts; numbers in overlap regions indicate shared orthologous transcripts; numbers in the non-overlap regions represent the mapped orthologous transcripts unique to each species.

To verify the functional conservation and structural diversity of the BDGPs/BDTPs, we next to analyzed the functions and structures of orthologs of the 2166 maize BDTPs in the genomes of three other species related to maize, *Brachypodium distachyon, Selaginella moellendorfii,* and *Ostreococcus tauri*[27–29]. The unicellular green alga *Ostreococcus tauri* (Prasinophyceae) is an ancient member, which is the world’s smallest free-living eukaryote known to date, in the 1,500 million years old green lineage [27]. The *Selaginella moellendorfii* like all lycophytes which is an ancient lineage that diverged shortly after land plants evolved vascular tissues has features typical of vascular plants [29]. The *Brachypodium distachyon* is the first member of the Pooideae subfamily to be sequenced [28]. These three species could represent the important evolution point of plants and whose genomes have been sequenced to allow genomic analysis. We firstly analyzed the function and structure of the orthologs of the 2166 unique BDTs of 1696 maize BDTPs in the *Brachypodium distachyon, Selaginella moellendorfii*, and *Ostreococcus tauri* genomes, we found that the conservation of BDTP function and structure increased with closer relatedness to maize (Table 5). Then, we analyzed the function and structure of the orthologs of the 3884 unique BDTs of *Ostreococcus tauri* 2288 BDTPs in the *Brachypodium distachyon, Selaginella moellendorfii*, and maize genomes, to our surprise, the three genomes had almost identical numbers of orthologs but no BDTPs even with the threshold value for distance between TSSs set to 5.0 kb (Table 6). The two ways of analysis the orthologs of BDGPs/BDTPs between the four species show a common point that the number of orthologs is similar among the four species. To identify the common orthologs of the four species, we employ the Venn diagram to make a functional classification the orthologs in two ways which corresponding to the two ways of orthologs searching as Tables 5 and 6 shown. We firstly analysis the orthologs of 2166 maize BDTs in *Brachypodium distachyon*, *Selaginella moellendorfii* and *Ostreococcus tauri,* and the result shows that 726 common orthologs are shared by the four species (Figure 5A). Then, 1454 common orthologs can be found that are shared by the four species when we use 3884 *Ostreococcus tauri* BDTs to identify orthologous BDTs in *Brachypodium distachyon*, *Selaginella moellendorfii* and maize (Figure 5B).

**Table 5:** **Genes orthologous to maize BDTPs in three other plant species**

Species	Genome size (Mb)	Total	Distance between TSSs of gene pairs			
			0.5 kb	1 kb	2 kb	5 kb
***Brachypodium distachyon***	272	1714	586	656	774	978
***Selaginella moellendorfii***	212	1371	NA	15	28	60
***Ostreococcus tauri***	12.57	744	NA	NA	NA	NA

In total, 2166 unique BDTs from 1696 maize BDTPs were used to search for orthologous transcripts.

**Table 6:** **Genes orthologous to** ***Ostreococcus tauri*** **BDTPs in three other plant species**

Species	Genome size (Mb)	Total	Distance between TSSs of gene pairs			
			5 kb	10 kb	50 kb	100 kb
***Brachypodium distachyon***	272	1571	NA	1	9	18
***Selaginella moellendorfii***	212	1597	NA	2	5	9
***Zea mays***	2700	1573	NA	2	3	9

In total, 3884 unique BDTs from 2288 *Ostreococcus tauri* BDTPs were used to search for orthologous transcripts.

These results indicate that the function of the BDGPs/BDTPs is conserved but their structure is dynamic during the course of evolution. As a result, the structure of the BDGPs/BDTPs of *Ostreococcus tauri* has been separated by new genome sequences and that new species form their own BDGPs/BDTPs. However, the function of the orthologs is relatively conserved.

## Discussion

A single gene may produce several different transcripts, each with its own TSS and corresponding promoter. The locus of such a gene contains the coding regions for all of the transcripts, meaning that the outermost 5′ and 3′ boundary coordinates of the gene are defined by the most upstream TSS and the most downstream transcription termination site. All previous genome-wide searches for BDGPs adopted the search model created by Trinklein [5, 6, 17, 18] meaning that some BDGPs may have gone undetected. For this reason, we made a key modification to the search model based on the use of unique transcript loci instead of unique gene loci (Figure 1B). Our comparative study of five plant genomes confirmed that the modified search model generated more comprehensive data sets (Tables 1 and 2).

Two reports of genome-wide searches for BD PROs in plant species have been published. Dhadi et al. [17] focused on identification of BD PRO motifs based on bioinformatics-based predictions, but the promoter activities of the putative BD PROs were not verified experimentally. In another study, a hierarchical stochastic language model (HSL) was used to identify intergenic regions enriched for regulatory elements and considered to be BD PROs [18]. In our study, we combined bioinformatics analysis of microarray and RNA-seq data to conduct a genome-wide search and identify BDGPs. We also cloned a large number of putative promoters from the maize inbred line B73 and confirmed the biological activity of BD PROs using a transient expression system. We successfully detected BD PRO activity in 20 DAP maize embryos (Figures 2 and 3, and Additional file 4). We found that the ratio of confirmed BD PROs to putative BD PROs varied widely among the three types of data sets used to identify the putative BD PROs. The RS data set produced the highest ratio (11/18), which was significantly higher than those for the BD PROS identified based on the GS (20/129) and MA (3/9) data sets. Two factors may have contributed to this variation. First, the model searched for BDGPs based on predicted TSSs. This approach had a potential defect in that we cloned the intergenic regions as putative BD PROs based on the predicted TSSs. Thus the cloned promoter regions may not have contained the full 5′UTRs. An incorrectly predicted TSS could result in truncation of the conserved region of the full promoter, leading to reduced or even loss of promoter activity. Most of the putative GS BD PROs may have suffered such a truncation, leading to the low rate of recovery of active BD PROs. We cloned nine putative BD PROs based on the MA data set using the predicted TSSs. Three promoters showed activity in only one direction and the remainder exhibited no activity in either direction (data not shown). For this reason, we re-cloned the nine MA data set promoters using the ATG translation start site of each gene; three putative BD PROs that displayed bidirectional activity and five that showed promoter activity in one direction were recovered (MA-n without a red box in Figure 3A). We also re-cloned the 18 RS data set promoters using the ATG start site of each gene and recovered 11 putative BD PROs that functioned as BD PROs and 7 that showed promoter activity in one direction (RS-n without a red box in Figure 3B). The second factor that might have contributed to the variation in BD PRO recovery was tissue-specific expression of some putative BD PROs coupled with the transient expression system that involved use of embryos to assess BD PRO function. The set of putative BD PROs identified using the RS data set was derived from the intergenic regions of gene pairs which had relatively high expression values in 21- and 25-DAP embryos (Additional file 2). The putative GS BD PROs were not initially selected for functionality in embryos. The low recovery of BD PROs from among the nine putative BD PROs identified from the MA data set may have been due to false expression values. The microarray probes could not distinguish between different transcripts from a single gene, so relative expression values represented the combined expression of all transcripts from one gene rather than of one specific transcript.

Because of the limited size of their genomes, prokaryotic organisms employ efficient strategies to organize genes. Operons, overlapping genes, and nested genes are the typical forms of gene organization adopted by prokaryotic organisms [30]. The sharing of a promoter region by BDGPs can be considered one form of efficient gene organization. All sequenced higher plants, and human genomes are much larger than bacterial genomes, suggesting that compact gene organization is unnecessary in these higher organisms. However, several studies have shown that BDGPs are a type of compact gene organization that is a common architectural feature of the genomes of higher organisms [4–6, 17, 18]. Our study confirmed this phenomenon in eight plant genomes and suggests a potential evolutionary pathway for its origin. Moreover, the BDGPs are usually distributed in some pathways or cellular locations. Trinklein et al. [5] reported that the BDGPs function in DNA repair in human. Wang et al. [18] applied a strict criterion to identify functional associated bidirectional genes and found that most of them limited to several GO functions such as ATP binding et al. in *Arabidopsis*. Kourmpetli et al. [19] reported that some BDGPs were involved in seed development and related hormone/stress responses in *Arabidopsis*. Our results are consistent with these reports, DNA, RNA and nucleotide binding, and responses to stimulus are the common characteristics in all five plant species (Figure 4 and Additional file 5). These results indicate that the BDTPs have a tendency to display similar functions, and have a co-expression phenomenon base on the microarray data and RNA-seq data (Additional file 2). Studies about the organization and regulatory mechanisms of BDGPs in vertebrates pointed out that it seemed to be the cis-regulatory sequences in bidirectional promoters rather than the promoter length played the key role [5, 22]. Lin et al. gave an extensive study on the transcription factor binding sites in the human BD PROs and suggested that this was a simple mechanism for regulating genes by a limited set of TFs [31]. Krom and Ramakrishna also scanned 1, 16 and 39 regulatory elements overrepresented in intergenic regions of strongly correlated gene pairs in *populus*, rice and *Arabidopsis*, respectively [32].Wang et al. scanned the BD PRO region with HSL and assigned a transcriptional regulatory region (TRR) score. The results showed that most of the BD PROs with high TRR scores contain only one score peak which indicated that the regulatory mechanism of BDGPs may be the common regulatory elements taking effects cooperatively [18]. Dhadi et al. further confirmed this hypothesis in rice, *Arabidopsis* and *populus*, they identified a series of motifs overrepresented in BD PROs and speculated that those motifs may regulate expression of genes in both orientations through enhancer-like properties [17].

The location of the products of the BDGPs mainly in membrane and subcellular organelles for storage of nucleic acid, this result is consistent with Kourmpetli’s report [19]. However the location information indicated that the BDGPs may involve in gene expression regulation rather than function as structure components for the located subcellular organelles are used for storage of nucleic acid and the membrane is the scene of signal transduction.

Structural analysis of the orthologous BDTs revealed diversity among the plants based on evolutionary lineage (Tables 5 and 6), but the function of the BDTs is conserved among the plants (Figure 5). Hence, we can conclude that the function of the BDGs/BDTs is conserved in different species but that the BDGPs/BDTPs in new species are formed over a long evolutionary history rather than preserved as part of an ancestral heritage. The process of breaking and establishing BDGPs may be a necessary condition for the evolution of a new species. Investigation of the genomic organization of BDGPs in various species, especially key lineages in plant evolution, will be important for understanding the evolution of plant form and function.

## Conclusions

We developed an improved genome-wide search model for identifying BDGPs and showed that BDGPs are abundant in maize as well as in the genomes of five other plant species. We also used a maize embryo transient expression system to characterize the activity of a large number of maize BD PROs identified from three different data sets. Comparative analysis of the sequence characteristics of the BD PROs and the BDGP expression patterns in the five plant species showed that BDGPs are a common feature of plant genomes, and the analysis of function and structure of orthologous BDTs suggested their origin and a potential pathway for their evolution.

## Methods

### Identification of BDGPs

The genome annotation data of maize, sorghum, rice and soybean were downloaded from the PlantGDB (http://www.plantgdb.org/download/download.php?dir=/Sequence/xGDB). The genome annotation data of *Arabidopsis*, *Brachypodium distachyon* and *Selaginella moellendorfii* were downloaded from the Phytozome (http://www.phytozome.net/). The genome annotation data of *Ostreococcus tauri* was downloaded from the BEG (http://bioinformatics.psb.ugent.be/genomes/). We sorted the lists of gene and transcript location annotation data using the Python programming language. All gene and transcript locations were arranged by physical location from the 5′ end to the 3′ end of each chromosome. We filtered for gene pairs located on the same chromosome with an intergenic distance of less than 1.0 kb between the 5′ ends of the two genes; these were considered to be BDGPs.

### Promoter sequence analysis

The maize, sorghum, rice, soybean, and *Arabidopsis* genomic sequences [33–37] were downloaded from Phytozome (http://www.phytozome.net/). Based on BDTP annotation data, BD PRO sequences were collected from the genomic sequences using the Python programming language. RD PRO sequences were derived from 1000 random genes from each species. The RD PRO lengths were similar to the mean length of the BD PROs from each species. The GC content of the RD PROs was calculated as the mean of 20 random data sets. Four conserved TATA-box sequence motifs (TTATTT/TATAAAT/TATTAAT/TATATAA) were detected in the approximately −10 to −50 region at both ends of the BD PROs.

### Functional assays of BD PROs

We screened over 500 BD PROs ranging from 50–1000 bp in length for those lacking *Xba*I and *Nco*I restriction enzyme sites for use in the dual reporter gene (GUS/GFP) vector pBD68GG3. *Xba*I and *Nco*I restriction sites were added to the 5′ ends of forward and reverse primers, respectively. PCR products digested with *Xba*I and *Nco*I were substituted for the intergenic region between the GUS and GFP genes in the pBD68GG3 vector. In total, 129 BD PROs were cloned and used in a transient expression assay (a list of primers is provided in Additional file 2).

We successfully cloned 9 and 18 BD PROs based on microarray data for the maize inbred line B73 (GEO accession number: GSE54310) and RNA-seq data (NCBI SRA accession number: SRA060791; SRA035621) (list of primers is available in Additional file 2), respectively, that resulted in high gene expression in 20 DAP embryos. The BD PROs were inserted into pBD68GG3 using the cloning strategy described above.

We prepared plasmid DNA (QIAGEN) for each construct and used 1 μg of plasmid DNA per transient transformation carried out by particle bombardment. Each construct was used in two bombardments with six embryos per bombardment. Constructs containing GFP and GUS fusions were initially tested in immature maize embryos (20 DAP immature maize embryos grown in the greenhouse) using a Bio-Rad (Bio-Rad Laboratories, Hercules, CA, USA) gene gun following standard protocols. GFP expression in immature maize embryo was detected 18–24 h after bombardment using a Zeiss Axioskop epifluorescence microscope (Carl Zeiss MicroImaging Inc, Thornwood, NY, USA) using GFP filter sets, a 5 × field lens, and the Axiovision image analysis software. Histochemical staining for GUS activity was performed as described previously [38] following completion of GFP fluorescence detection.

### Blast2GO suite bioinformatics analysis

Protein sequences translated from the BDTs of each species were analyzed using the Blast2GO bioinformatics software suite enabling us to make Combined Graphs using some parameters. We choose the most strict sequence filter value (only nodes in which the sequences number more than 10% the total amount of sequences) and a relatively strict node score filter value (>100) to make the last Combined Graphs.

### Identification of orthologous BDTs

We used BLASTp software to search for transcripts orthologous to the 2166 maize BDTs in the sorghum, rice, soybean, *Arabidopsis*, *Brachypodium distachyon*, *Selaginella moellendorfii*, and *Ostreococcus tauri* genomes (e-value < 10^−10^). We also used the BLASTp software to search for transcripts orthologous to 3884 *Ostreococcus tauri* BDTs in the *Brachypodium distachyon*, *Selaginella moellendorfii*, and *Zea mays* genomes. We calculated the distances between the TSSs of the mapped orthologous transcripts that were arranged head-to-head on the same chromosome and sorted them based on thresholds for the distance between the TSSs of mapped orthologous transcripts.

### Availability of supporting data section

A maize microarray expression data set: (GEO accession number: GSE54310).

Two RNA-seq data sets: (NCBI SRA accession number: SRA060791; SRA035621).

The other supporting data are included as additional files.

## Electronic supplementary material

Additional file 1: **List of location annotations for BDGPs/BDTPs of five plants.** (XLSX 1 MB)

Additional file 2: **List of expression annotations for BDGPs/BDTPs of maize and BD PROs clone primers.** Worksheet “expression profile”: Analysis was repeated three times per tissue; R: BDG in the reverse strand; F: BDG in the forward strand. Worksheet “candidate”: Y-axis: relative expression values of candidate BDGs; X-axis: B73 maize tissues, E: Embryo; En: Endosperm; R: BDG in the reverse strand; F: BDG in the forward strand. Worksheet “RNA-seq”: #N/A: No transcript detected; R: BDT in the reverse strand; F: BDT in the forward strand. A BDGP that produced more than two transcripts would result in more than one BDTP. This would lead to cloning of more than one putative BD PRO derived from a single BDGP. To remove such redundant promoters, we sorted the putative promoters that were derived from one BDGP by length and then retained the putative promoters that were at least 50 bp longer than the shorter promoters. Hence, we annotated 814 unique BDTPs that contained unique putative maize BD PROs. Worksheet “primers”: b–n: Used to clone promoters identified using the MA data set; 7-n: Used to clone promoters identified using the RS data sets. 1-n/5-n: Used to clone promoters identified from a genome-wide search (GS). (XLSX 2 MB)

Additional file 3: **Distribution of BD PRO lengths, BDTPs in each chromosome and similar types of gene organization in the genomes of five plant species.** Worksheet “chromosome”: A: maize; B: sorghum; C: rice; D: soybean; E: Arabidopsis; Chr: chromosome; Mt: mitochondrion; Pt: plastid; Sy: fgenesh gene; Un: location has not yet been defined. Worksheet “organization”: The rate of gene pairs is the total number of genes of one type of gene organization compared to the total number of genes of each species. BDGPs: the distance between the 5′ ends of the two genes was less than 1.0 kb; CPGs: the distance between the 3′ ends of the two genes was less than 1.0 kb; CDGPs-F: the distance between the 3′ end and the 5′ end of the two genes on the forward strand was less than 1.0 kb; CDGPs-R: the distance between the 3′ end and the 5′ end of the two genes on the reverse strand was less than 1.0 kb. (XLSX 2 MB)

Additional file 4: **Identification of putative GS BD PROs by transient expression of GFP and GUS in immature maize embryos.** Putative GS BD PROs showed either promoter activity in one direction or no promoter activity. CK: Embryos bombarded with empty vector plasmid using a particle gun. (XLSX 19 MB)

Additional file 5: **Classification of the characteristics of the BDTs of four plants.** Worksheet “CC”: A, C, E, and H: Multilevel pie charts of the CCs for sorghum, rice, soybean, and Arabidopsis, respectively. B, D, F, and I: Combined graphs of the CCs for sorghum, rice, soybean, and Arabidopsis, respectively. Worksheet “MF”: A, C, E, and H: Multilevel pie charts of the MFs for sorghum, rice, soybean, and Arabidopsis, respectively. B, D, F, and I: Combined graphs of MFs for sorghum, rice, soybean, and Arabidopsis, respectively. Worksheet “BP”: A, C, E, and H: Multilevel pie charts of the BPs for sorghum, rice, soybean, and Arabidopsis, respectively. B, D, F, and I: Combined graphs of BPs for sorghum, rice, soybean, and Arabidopsis, respectively. (XLSX 7 MB)

## Abbreviations

MF: Molecular functions
CC: Cellular components
BP: Biological processes
TSS: Transcription start site
DAP: Days after pollination
BDGP: Bidirectional gene pair
BDG: Bidirectional gene
BDTP: Bidirectional transcript pair
BDT: Bidirectional transcripts
BD PRO: Bidirectional promoter
RD PRO: Random polar directional promoter
MA: Microarray
RS: RNA-seq
GS: Genome-wide search
CGP: Convergent gene pair
CDGP: Co-direction gene pair.
